# Barriers and outcomes of therapeutic communication between nurses and patients in Africa: a scoping review

**DOI:** 10.1186/s12912-024-02038-0

**Published:** 2024-05-30

**Authors:** Susanna Aba Abraham, Frederick Nsatimba, Dorcas Frempomaa Agyare, Joyce Agyeiwaa, Rita Opoku-Danso, Jerry Paul Ninnoni, Patience Fakornam Doe, Benjamin Osei Kuffour, Benjamin Kofi Anumel, Gifty Osei Berchie, Christian Makafui Boso, Andrews Agyei Druye, Christiana Okantey, Gifty Owusu, Paul Obeng, Mustapha Amoadu, Isaac Tetteh Commey

**Affiliations:** 1 Department of Adult Health, School of Nursing and Midwifery, University of Cape Coast, Cape Coast, Ghana; 2 Department of Mental Health, School of Nursing and Midwifery, University of Cape Coast, Cape Coast, Ghana; 3 Department of Maternal and Child Health, School of Nursing and Midwifery, University of Cape Coast, Cape Coast, Ghana; 4 Department of Health, Physical Education and Recreation, University of Cape Coast, Cape Coast, Ghana

**Keywords:** Barriers, Outcomes, Therapeutic communication, Nurses, Patients, Africa

## Abstract

**Background:**

Therapeutic communication (TC) promotes effective patient care, emotional wellbeing, and improves patient outcomes. The purpose of this review was to synthesise evidence on barriers and outcomes of TC between nurses and patients in Africa and to inform future studies and systematic reviews.

**Methods:**

Search for the records was done in four major databases including PubMed, Science Direct, PubMed CENTRAL, and JSTOR. Additional searches were done in Google Scholar and Google. Results and findings from published studies and grey literature were included. A total of 16 articles met the eligibility criteria and were included in the review. This scoping review followed the guidelines by Askey and O’Malley.

**Results:**

Barriers associated with TC were grouped under five main themes: sociodemographic factors, patient-related factors, nurse-related factors, environmental and health service-related. Age, and religious beliefs were the main sociodemographic factors that hindered TC while stress and inadequate knowledge and skills were identified among the nurse-related barriers to TC. Extreme weather conditions and mosquito infestation were environmental factors while lack of access to TC educational programmes on TC was a health service-related factor that interfered with TC. Both positive and negative outcomes of TC were also identified.

**Conclusion:**

Continuous professional development on TC is necessary to improve nurses’ attitudes and update their skills to enable them to render culturally competent nursing care to patients.

**Supplementary Information:**

The online version contains supplementary material available at 10.1186/s12912-024-02038-0.

## Introduction

Therapeutic communication (TC) is fundamental to health service delivery [1]. TC includes the usage of communication and the development of a professional relationship between the nurse and the patient which may lead to positive patient outcomes that promote healing and recovery [2]. Therapeutic communication has evolved significantly within the nursing profession, reflecting an increasing understanding of its importance in patient care [1]. Initially rooted in basic communication principles, TC has matured to encompass a holistic approach that considers patients’ emotional needs, cultural backgrounds, and individual preferences [2]. Over time, nursing knowledge has recognized TC as not just a tool for relaying information but as a means to establish trust, empathy, and a supportive environment conducive to healing [3]. This evolution has been marked by a shift towards patient-centered care models, where nurses actively engage in dialogue, active listening, and empathetic responses to better address patient concerns and foster collaborative relationships [2, 3]. As nursing continues to advance, TC remains a cornerstone, adapting to new technologies, diverse patient populations, and emerging healthcare challenges to ensure the delivery of compassionate and effective care.

Therapeutic communication, when practised consistently and effectively, has the potential to promote effective patient care, emotional wellbeing, and improve patient outcomes [3]. Bright and Reeves claimed that the art of TC entails prioritising patients’ needs and fostering a supportive environment in which care can be provided and accessed [4]. It also involves employing various interpersonal skills such as empathy, understanding and active listening which aims at establishing a meaningful connection with patients [5].

Navigating through the health system can be a daunting task for most patients and clients who may experience uncertainty and a sense of vulnerability [6]. In spite of this challenge, nurses are in a central position to create a supportive and welcoming atmosphere for these patients. A positive nurse-patient interaction is said to contribute to positive patient experiences and satisfaction, and better treatment outcomes [2, 7]. Thus, nurses who spend longer times with patients are required to maintain a therapeutic relationship with clients and apply therapeutic communication to explore their physical and emotional well-being [8].

However, following decades of research on TC, there is a lack of review study that has mapped evidence on outcomes and barriers to effective TC [9]. This is typical of Africa where inadequacies are reported in the utilisation of TC which impacts negatively on the provision of nursing care [9, 10].

It is argued that medical errors and patient injuries can be decreased with effective use of TC [4] which may also promote the psychosocial wellbeing of the client [11]. This review will fill this gap by synthesising the evidence on barriers and outcomes of TC between nurses and patients in Africa. The purpose is to inform future studies and systematic reviews. Additionally, this review aims to furnish substantiated evidence that has the potential to enrich the creation of policies and the integration of approaches geared towards enhancing therapeutic communication within nursing education and practice.

## Methods

This scoping review is conducted using the guidelines by Arksey and O’Malley [12]. The steps include specifying the research question, identifying relevant literature, selecting studies, mapping out the data, summarising, synthesising, reporting of the results and including expert consultation. The reporting of the review follows PRISMA-ScR specifications [13].

Research questions which formed the basis for the review included: (1) What are the barriers of TC among nurses and patients in Africa (2)? What are the outcomes of TC between nurses and patients in Africa?

The inclusion and exclusion criteria for the review are presented in Table 1.

**Table 1:** Eligibility criteria

Inclusion criteria	Exclusion criteria
1. Studies published in 2010 and later 2. Refereed or peer reviewed articles, original and grey literature 3. Papers published in English language 4. Articles conducted on TC 5. Studies conducted in Africa	1. Studies conducted in other languages other than English Language 2. Studies conducted before 2010 3. Conferences papers and commentaries 4. Studies on pre-service nursing personnel 5. Studies on auxiliary nurses

Medical Subject Headings (MeSH) terms were used for the search in PubMed and subsequently modified for search in other databases (Science Direct, PubMed CENTRAL, and JSTOR). Table 2 shows the keywords and MeSH terms used for the strategic search in PubMed.

**Table 2:** Keywords and MeSH terms

Search (#)	Search terms
#1 Search to identify the barriers to TC	Barriers*[MeSH Terms] Barriers* OR Hinderance* OR Limitations* OR Obstacles* OR impediments* OR Challenges* OR Constraints* OR Difficulties*
#2 Search to identify the TC	Therapeutic communication* [MeSH Terms] OR Empathic communication* OR Active listening* OR Compassionate communication*
#3 Search to identify the nurses	Nurses*[MeSH Terms] OR Midwives* OR Registered Nurses* OR Professional nurses* OR nursing staff* OR mental health nurse*OR psychiatric nurse* OR nursing personnel* OR Licence practical nurse*
#4 Search to identify outcomes of Therapeutic Communications between nurses and patients	Outcomes* [MeSH Terms] OR Positive Outcomes* OR Favourable health results* OR Beneficial health effects* OR Good health consequences* OR Successful health outcomes* OR Optimal health impacts* OR Desirable health outcomes* OR Productive health results* OR Satisfactory health outcomes* OR Wellness improvements* OR negative Outcomes* OR Adverse health effects* OR Detrimental health consequences* OR Harmful health consequences* OR Unfavourable health repercussions* OR Poor health impacts* OR Undesirable health effects* OR Detrimental health outcomes* OR Detrimental health progress*
#5 Search to identify the Sub-Saharan Africa	Africa* [MeSH Terms] OR Central Africa * OR Eastern Africa* OR Southern Africa* OR Western Africa* OR Northern Africa* OR Algeria* OR Angola* OR Benin* OR Botswana* OR Burkina Faso* OR Burundi* OR Cape Verde* OR Cameroon* OR Central African Republic* OR Chad* OR Comoros* OR Congo* OR DR Congo* OR Cote d’Ivoire* OR Ivory Coast* OR Djibouti* OR Egypt* OR Equatorial Guinea* OR Eritrea* OR Eswatini* OR Swaziland* OR Ethiopia* OR Gabon* OR Gambia* OR Ghana* OR Guinea*OR Guinea-Bissau*OR Kenya* OR Lesotho* OR Liberia* OR Libya* OR Madagascar* OR Malawi* OR Mali* OR Mauritania* OR Mauritius* OR Morocco* OR Mozambique*OR Namibia* OR Niger* OR Nigeria* OR Rwanda* OR Sao Tome and Principe* OR Senegal* OR Seychelles* OR Sierra Leone* OR Somalia* OR South Africa* OR South Sudan* OR Sudan*OR Tanzania* OR Togo* OR Tunisia* OR Uganda* OR Zambia* OR Zimbabwe*
#5 Overall Search strategy Filters activated	#1 AND #2 AND #3AND #5 NOT animal* #1 AND #2 AND #3 AND #4 AND #5 NOT animal* English language Date (01/01/2010-14/03/2023)

Additional searches were conducted in Google Scholar and Google for relevant materials. Records were exported into a Bibliographic Manager (Mendeley) and duplicates removed. Articles which were not relevant to the subject matter were also excluded. Reference check and institutional repository search also revealed some records which were screened by the team. Furthermore, reference lists of all eligible articles were carefully checked for potentially relevant full text articles. Complete articles that met the eligibility criteria for the review were finally saved in Mendeley for data extraction and charting.

Data extraction was conducted independently by two groups comprising six members each. To retrieve relevant studies, FN, JA, DFA, ROD, SAA, ITC, BKA, BOK, independently extracted the data for analysis. Details such as authors, the countries in Africa where the study was conducted, the purpose of the study, study design, population, sample size, study findings, and conclusions were extracted. Data were compared and inconsistencies that were detected from the charting reconciled by independent team members (MA, FN, JA, DFA, ROD, SAA, ITC, BOK). Thematic analysis and data synthesis were carried out using the method proposed by Braun and Clarke (2006) and the results presented according to the research questions. Authors acquainted themselves with the data that was extracted, and codes were assigned to the data to aid in the content's description. The gathered data was then examined by the authors for patterns and themes. The developed themes were then examined, described, and identified, and the outcomes presented. The review started on June 13 and ended with the last search on August 18, 2023.

### Search results

**Fig. 1:**
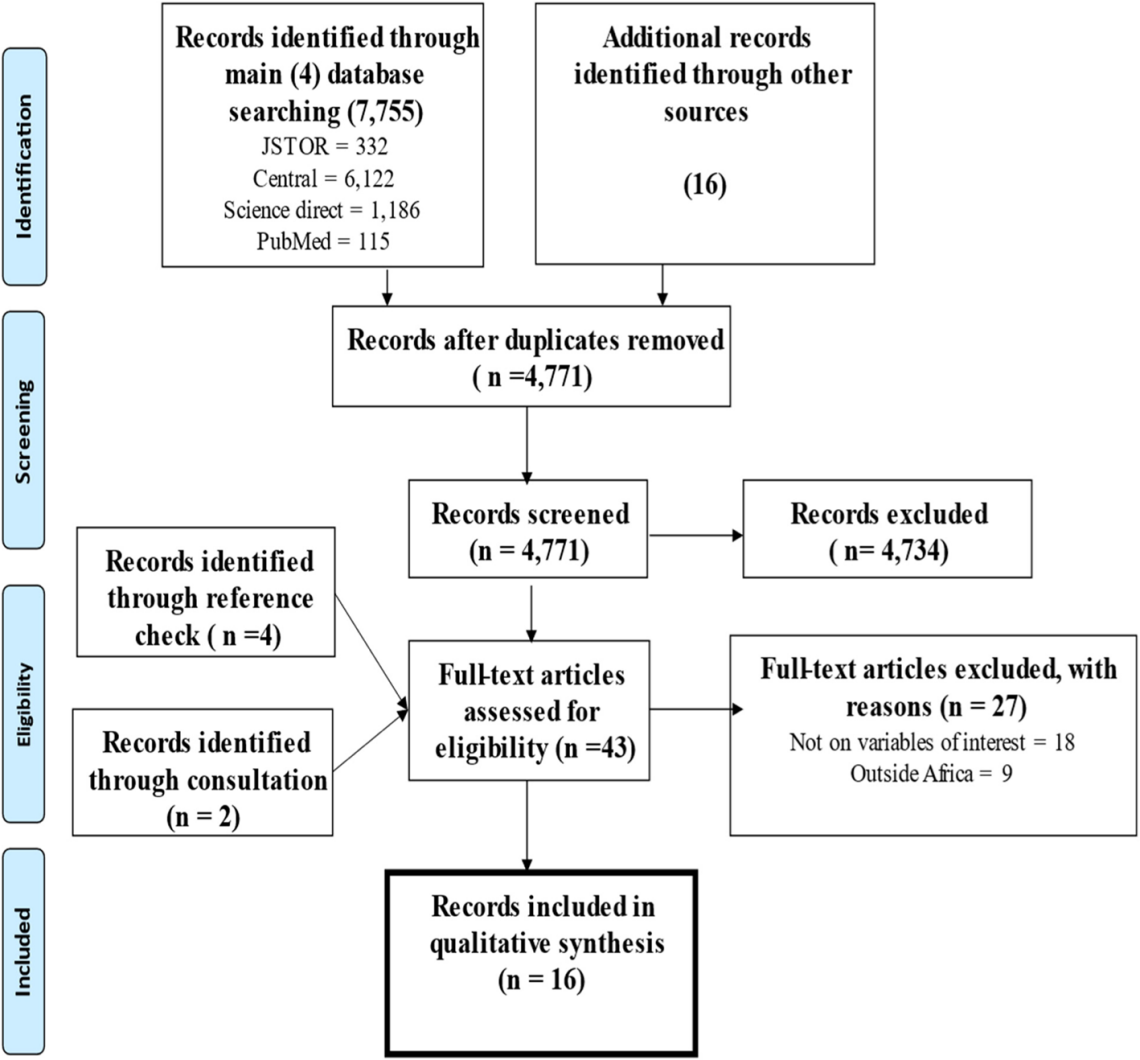
PRISMA Chart

A total of 7,755 records were retrieved. Sixteen records were retrieved from additional search in other databases (Google Scholar and Google). A total of 3,000 duplicates were removed. Following title and abstract screening, 37 full-text records remained after excluding 4734 records. The excluded records at this phase were not relevant to the topic under review. Checking the reference list of all the full-text records produced four records while consultation with a digital librarian also produced 2 records. A total of 16 records were finally included in this review. Reasons for excluding full-text records were provided. Details of search results and screening process are presented in Fig. 1.

### Study characteristics

**Fig. 2:**
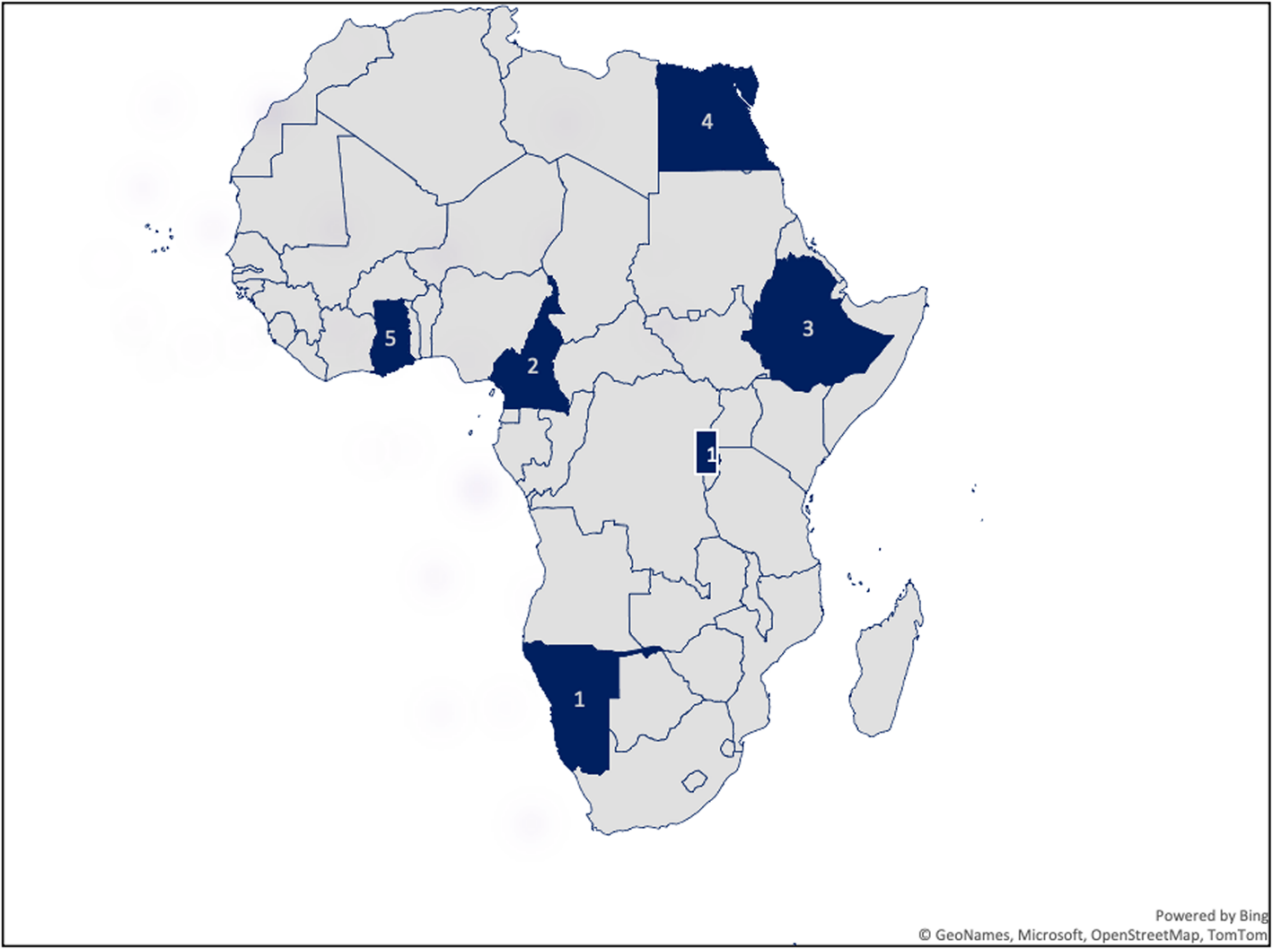
Map showing countries from which reviewed studies were conducted

**Fig. 3:**
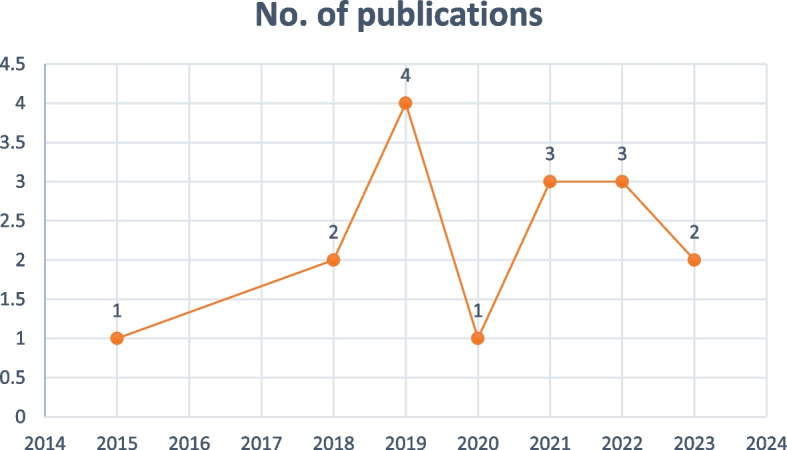
The years in which the studies were published

**Fig. 4:**
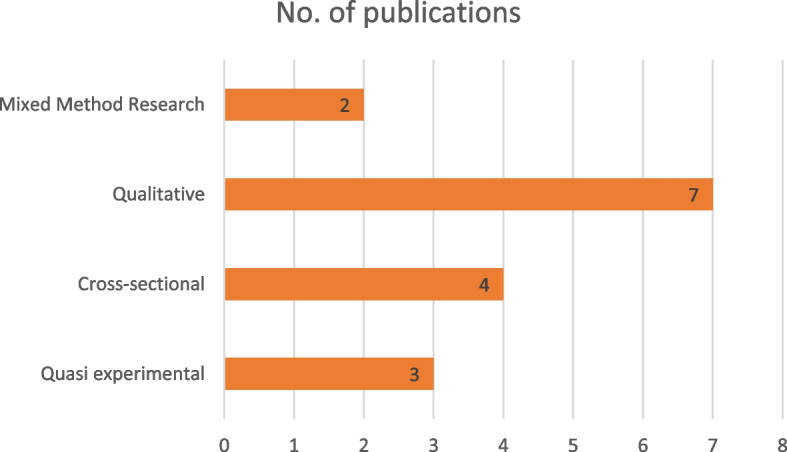
Study designs of reviewed studies

Most reviewed studies used a qualitative design followed by cross-sectional designs with a few using mixed method (see Fig. 3). Majority of the reviewed studies were conducted in three countries in Africa including Ghana, Egypt and Ethiopia. See Fig. 2 for details. Also, most of the studies were conducted in 2019. Figures 3 and 4 present the years in which the studies were published and study designs respectively.

### Findings

Findings were reported according to research questions (Barriers associated with TC and outcomes of TC).

#### Barriers

Barriers associated with TC were grouped into sociodemographic factors, patient-related factors, nurse-related factors, environmental and health service-related. The outcomes were grouped under positive and negative outcomes of TC (Table 3).

**Table 3:** Themes and sub-themes

Main Themes	Specific Barriers	Author
**Sociodemographic characteristics**	Old age of patients	[14–17]
	Younger age of patient & nurses	[14–16, 18]
	Male Muslim prefers males and vice versa	[14]
	male patients don’t prefer female patients and vice versa	[15–17]
	younger male patients prefer female patients and vice versa	[16]
	Being single (not married)	[16]
	Religious belief and tenets	[14, 15]
	Cultural differences and practices	[14, 15]
	Beliefs in witchcraft of dying older patients	[15]
	Patients with Formal education	[17]
	Limited literacy of patients	[19]
	All knowing patients	[14, 15]
	Language differences	[16, 17, 19]
**Nurse-related barriers**	Nurses Stress	[20, 21]
	Few years of working experience	[19]
	Longer years of practice (> 10 years)	[22]
	Resistance and reluctance of the nurse to communicate with patients	[19]
	Nurses’ inadequate knowledge & skills on therapeutic communication	[14–17, 19, 21, 23]
	Use of jargons/technical terms by the nurse	[17, 19]
	Lack of access to therapeutic educational programme.	[17, 18, 24, 25]
	Inability of nurses to explain technical terms	[15]
	Negative nurses’ attitude and behaviour,	[20, 26]
	Nurses Lack of empathy from	[15]
	Lack of professional decency & urgency of nurses	[15]
	Lack of love for the job	[27]
	Oppressive or judgmental language	[23]
	Nurses’ personality or mood	[23]
	Unwillingness to work in poor working environment	[27]
**Patient-related barriers**	Negative attitude of the patient towards the nurse	[19]
	Negative attitude of patients’ relatives.	[15, 17, 26]
	Patient’s dissatisfaction with nurses	[14, 15]
	Idleness	[14]
	Misconceptions about nurses	[14]
	Stereotyping,	[15]
	Lack of empathy,	[15]
	Lack of respect from the nurses	[15]
	Bad nursing care experience	[23]
	Pain	[14–17, 19, 28]
	Anxiety	[15, 19]
	Physical discomfort of the patient	[19]
	Deafness,	[26]
	Patient condition	[20]
	Patient mood swings	[27]
	Perceived lack of confidentiality	[19]
	Patients’ family interference with care	[16, 17, 20]
	Power imbalance	[23]
**Environment-related barriers**	New environments	[14, 19]
	Long stay in health facilities	[14, 15]
	Hot and cold wards	[14, 15, 19]
	Distracting light in wards	[14, 19]
	Noisy environment	[14–16]
	Fan in the ward	[14]
	Busy nature of the ward environment	[16, 19]
	Mosquito infestation in the health facility	[14, 15]
	Poor hospital environment	[28]
**Health facility-related barriers**	Nurses’ shortage	[16, 21]
	High workload	[14, 17, 19–23, 28]
	Lack of access to educational programme on TC	[17, 18, 23–25]
	Lack of medical facilities and drugs	[16]
	Poor information on ward routines/orientation	[21]
	Ineffective health insurance	[16]

##### Sociodemographic barriers

The studies reviewed indicated that the age of patients affected TC. Older and younger age of patients were observed to influence the effectiveness of TC between nurses and patients [14–16, 18]. For instance, communicating with children requires the use of different words and strategies compared to adults since children cannot think in abstract terms. Emishaw also found that younger males preferred female nurses and vice versa [16].

Other studies reported that religious beliefs and tenets of both patients and nurses acted as barriers to effective TC in health facilities [14–16]. For instance, Muslim males preferred to be cared for by male nurses and vice versa [14]. In relation to gender, some studies found that therapeutic communication was effective when male patients were nursed by female patients and vice versa [15, 16]. Cultural differences and practices between nurses and patients also affected effective TC [14, 15]. For instance, beliefs that older dying patients had witchcraft affected TC between nurses and older patients [15]. Given that different cultures use language differently, language differences between nurses and patients were found to affect TC [16, 17, 19]. Therefore, ongoing validation is important in a therapeutic relationship to confirm interpretation of words. Another socio-demographic characteristic that impacted effective TC was education. Formal education on the part of patients was found to negatively affect the practice of TC in healthcare facilities [17]. Limited literacy of patients [23] was reported to affect TC. In addition, all-knowing attributes of some patients were more likely to impact negatively on TC between nurses and patients [14, 15]. Furthermore, a review study found that marital status and being unmarried was a barrier to TC [16].

##### Nurse-related barriers

Evidence from the review showed that stress among nurses was a barrier to TC [20, 21]. Also, a few years of work experience of the nurses negatively influenced TC [19]. Another study having more years of experience affected TC practice [22]. Inadequate knowledge and skills of TC [14–17, 19, 21, 23] was found to be a barrier. The use of technical terms and jargons [17, 19], and the inability of the nurses to explain technical terms [15] were also identified as barriers to TC. The review found that negative nurses’ attitudes and behaviours [15, 20, 26], lack of empathy [15], oppressive and judgemental attitudes [23] hindered TC between nurses and patients. Also, dissatisfaction and lack of love for the job [27], resistance and reluctance of the nurses to communicate with patients [19] impeded TC. The review found that the personality and/or mood of nurses hindered TC [23]. Furthermore, a study reported that the lack of professional decency and urgency of nurses was a barrier [15]. In addition, the unwillingness of nurses to work in poor working environments affected their communication with patients [28].

##### Patient related barriers

Evidence from this review suggested that, negative attitude of the patient towards the nurse was a barrier to TC [19]. In addition, the negative attitude of patients’ relatives hindered TC [15, 26].

Patient’s dissatisfaction with nurses was reported by [14, 15] as a hindrance to TC. Idleness on the part of the patient was identified as a barrier [14]. Also, patients’ misconceptions about nurses [14], stereotyping, lack of empathy, lack of respect from the nurses [15], and bad nursing care experience [23] were all found to affect TC between patients and nurses. The review also reported that pain [14, 16, 17, 19, 23], anxiety [15, 19], and physical discomfort of the patient [19], deafness [26], patient condition [20] and patient mood swings [27] were all identified as barriers to TC. Lack of confidentiality was identified as a barrier to TC [19]. Additionally, patients’ family interference with care also affected TC [16, 17, 20]. Cubaka et al. reported that power imbalance weighing on the provider’s side was reported to affect TC [23].

##### Environment-related barriers

The reviewed studies reported that anxiety related to new environments [14, 19] and long stay in health facilities [14, 15] negatively impacted TC between nurses and patients. Also, extreme weather events such as very hot and cold wards affected TC [14, 19]. Furthermore, noisy environments [14–16], distracting environments in relation to the light and fan [14, 19], and the busy nature of the ward environment [16, 19] also hinders the practice of TC. Also, mosquito infestation [14, 15] and poor environment of the health facility [28] also impacted TC between nurses and patients.

##### Health facility related barriers

In relation to health facility factors, shortage of nursing staff [16, 21] and high workload [14, 17, 19–23, 28] were identified as hindrances to TC. Additionally, lack of access to therapeutic educational programmes [17, 18, 23–25], lack of medical facilities and drugs and lack of continuous TC opportunities [16] were reported as barriers to TC. Other impediments to TC identified from the reviewed studies were poor information on ward routines [21] and ineffective health insurance system [16].

### Outcomes of therapeutic communication

The outcomes of TC between nurses and patients were grouped into positive and negative.

#### Positive outcomes

This review found that effective TC resulted in adherence of patients to treatment [21, 27]. Furthermore, effective TC improved patients’ satisfaction with treatment protocol [25], communication with nurses [22], instructions and acceptance of guidance from nurses [27]. Another study reported effective TC fostered patients’ trust and improved recovery [18]. Successful TC also resulted in improvement in nurses’ skills in TC and the quality of nursing care [24] protection of patients’ rights [22].

#### Negative outcomes

A reviewed study showed that ineffective TC contributed to poor treatment compliance, medical errors, patient dissatisfaction with care and inefficient use of resources [20]. Also, negative TC reportedly resulted in non-supportive management [21].

## Discussion

The aim of the scoping review was to map evidence on the barriers impacting the practice of effective TC and its outcome. Findings from this review suggest that there are numerous obstacles to effective TC between nurses and in-patients in Africa. These hindrances were clients’ sociodemographic characteristics such as age and religious differences, limited experience of nurses, patients’ negative attitudes, bad experience of nursing care, and physical discomfort. Other obstacles to TC which were identified in the review related to the environment, climate and the working conditions. These included; hot and noisy ward environments, lack of access to therapeutic educational programmes, shortage of staff and ineffective health insurance system. From the review, it was found that one of the barriers that negatively impacts TC is the shortage of nurses who provide direct patient care to in-patients [16, 21]. Nursing staff shortage could result in high workload for nurses who remain at work and therefore impede the ability to effectively communicate with patients. Consequently, this review also found high workload as an obstacle for effective TC between nurses and in-patients [14–17, 20, 22, 23]. Furthermore, the review indicated that job-related stress among nurses served as a catalyst for ineffectiveness of TC as the emotional and social elements of communication are lost [29]. There is therefore the need for healthcare administrators and nurse managers to address these healthcare related factors that impede TC between nurses and patients. Kwame and Petrucka suggest a thorough examination of how this could be addressed [9].

Furthermore, this review found negative nursing attitudes such as lack of empathy hindered effective communication with admitted patients [15]. Sharafkhani et al. reported that nurses’ inability to express empathy and accept their own emotions produces gaps in therapeutic communication [30]. This is unfortunate as empathy is central to effective TC in healthcare settings. Consequently, this lack of empathy can result in increased patient anxiety, hostility and poor clinical outcomes [31].

Another barrier to TC found by this review was limited practical experience of nurses [19]. A similar finding. Also, ineffective TC by trained nurses may serve as a negative reinforcement for preservice nurses and thus, cause the problem to become institutionalised [32]. There is the need for supportive supervision by nurse managers and supervisors to ensure that TC is appropriately practised during nursing service delivery.

Cultural beliefs and practices such as the notion that older dying patients were witches and wizards caused reluctance among nurses in effectively communicating with older dying patients [14, 15]. Most of these cultural barriers are compounded by the language differences that affect the effective communication of needs and expectations between nurses and patients [9, 14].

The inadequate knowledge and skill of nurses with regard to TC can have a profound impact on the quality-of-care patients receive and their overall outcomes [8]. TC is an essential element in the nursing profession that entails proficient engagement and interaction with patients to foster recovery, confidence, and comprehension [2]. The absence of the requisite knowledge and skills in this domain among nurses may result in various limitations and consequences including fatal medical errors. Access to training opportunities in TC for nurses plays a pivotal role in the development and enhancement of therapeutic engagement with patients [15, 19]. In turn, this can substantially ameliorate patient care and overall healthcare outcomes. The inability of nurses to explain technical terms and the use of medical jargon can equally have a significant impact on TC with patients [33]. Effective communication in healthcare requires clear and understandable dialogue between healthcare providers and patients. If nurses opt to employ intricate medical terminology that may not be comprehensible to patients, it may impede effective TC [14].

This review also identified positive outcomes of effective TC between and patients in Africa. Evidence suggested that successful TC leads to patient adherence [21, 27] and satisfaction [25] to treatment protocol these is in agreement with Sherrill et al. who reported that effective TC enables healthcare workers to actively explain and listen to patients and their families making it easier for them to understand their specific needs, preferences and concerns. The results are also similar to that of Abrams et al. who reported that families of patients are very satisfied with the communication made by nurses [34]. In addition, effective TC also leads to patients understanding instructions and guidance from nurses [27]. Risna & Fauzia reported that nurse TC had a positive effect on the cooperative behaviour of patients with mental disorders [35]. Hence, the patient developed trust which eventually leads to improved outcome [18]. Finally, the reviewed studies found that effective communication is essential in preventing medication errors [2, 8, 36].

### Limitations of the review

A significant majority of the included studies were qualitative and non-experimental. Included studies relied on self-report measures. This introduces biased responses from participants which may affect the generalisation of their findings. This situation may affect the generalisation of findings of this review.

Furthermore, the literature search was restricted to peer-reviewed articles and papers published in English. This situation may have affected the number of included studies and the depth of information presented in this review. Although the study focused on Africa, the majority of the papers were conducted in sub-Saharan Africa (SSA). This may be another limitation to generalising the findings to cover the entire African continent.

There could be papers unpublished on TC in languages other than English which may help understand TC’s importance and barriers in creating a safe and healthy work environment for healthcare delivery. However, this review focused primarily on papers published in English. This may reduce the number of included studies conducted on the subject matter in Africa.

Though there were limitations to the study findings, the review article is still relevant to nurses -patients relationship regarding TC.

### Recommendations for future research

Reviewed papers on TC mainly conducted in Africa were included in this study. Most of these papers were conducted using a qualitative approach with much attention on sub-Saharan Africa. Quantitative designs may be needed to understand the concept of TC in nursing care.

Additionally, present literature on the subject matter focuses on nurses and patients. Hence, studies that seek to explore views of physicians, surgeons, clinical psychologists, pharmacists, laboratory technicians and other auxiliary staff in healthcare facilities are needed.

Moreover, the direct impact of TC on nursing care may need further exploration. More studies would also be required to explore the strengths and weaknesses of TC in health care delivery in Africa. Finally, understanding TC through the experiences of other health workers in the health facilities will be of significant benefit for patient care.

### Implications for nursing practice, education and policy

The barriers for effective TC between nurses and patients in health care settings are numerous and require a comprehensive strategy to address them. Nurses who lack skills to practise TC might struggle to engage the patients at an emotional level. Hence, it leads to lower patients’ satisfaction levels. Also, TC must be integrated in all aspects of the nursing curricula to enhance nurses’ ability to effectively communicate with patients, families and healthcare teams. Policies to institutionalise supportive supervision on nurses practice of TC while interacting with patients should be established and implemented.

Cultural differences also hinder TC and affected nurses’ willingness to provide care to older dying patients. This means that geriatric patients may suffer neglect in health facilities while on admission. This may result in missed opportunities to provide comfort to dying patients, exacerbate patient anxiety and negatively impact patient care experience. Strategies to incorporate cultural competency and reflective practice in nursing practice should also be enforced.

Furthermore, shortage of nurses providing direct patient care results in increased workload. This could invariably cause stress among the nurses and affect the practice of effective TC negatively. When this is not resolved, it has the potential to impact preservice nurses who see these nurses as role models and mentors and serve as an avenue for negative learning. Health service managers must ensure the appraisal of nursing tasks and responsibilities and recruit more nurses to enhance opportunities for the implementation of effective TC.

Lack of access to educational programmes on TC may result in ineffective practice of TC such as using jargon and technical terms while communicating with patients. This could result in failure to effectively educate patients and families, and fester misunderstanding, anxiety and confusion. Nurse managers and health services administrators must invest in continuous professional development for nurses providing direct patient care to enhance their knowledge and skills.

## Conclusion

In conclusion, this comprehensive review illuminates the multifaceted nature of therapeutic communication (TC) within nursing practice and its pivotal role in patient care outcomes across diverse healthcare settings in Africa. The analysis underscores a myriad of barriers spanning sociodemographic, nurse-related, patient-related, environment-related, and health facility-related domains, all of which significantly impede the effective exchange of information, empathy, and support between nurses and patients. Notably, the shortage of nursing staff, high workload, and limited access to educational programs emerge as critical impediments to nurturing meaningful therapeutic relationships. Moreover, the review underscores the nuanced interplay of cultural beliefs, patient attitudes, and environmental factors that shape the landscape of TC, underscoring the imperative for culturally sensitive and patient-centered approaches in nursing care delivery. The findings also highlight the imperative for robust policies, comprehensive nursing education curricula, and supportive supervision mechanisms to foster a conducive environment for effective TC practices. Moving forward, there is a compelling need for future research endeavors to delve deeper into the quantitative dimensions of TC, explore diverse healthcare stakeholders’ perspectives, and elucidate the direct impact of TC on patient outcomes. Ultimately, by addressing the identified barriers and embracing a holistic approach to TC, nursing practice, education, and policy can collectively pave the way for enhanced patient satisfaction, improved treatment adherence, and optimized healthcare delivery across the African continent.

### Supplementary Information

Supplementary Material 1.

## Data Availability

All data generated or analysed during this study are included in this published article (and its supplementary information files).

## Abbreviations

TC: Therapeutic communication
MeSH: Medical Subject Headings
(SSA): Sub-Saharan Africa
