# Supplementary material for: Barriers and outcomes of therapeutic communication between nurses and patients in Africa: a scoping review
Source: BMC Nurs. 2024 May 30;23:362. doi: 10.1186/s12912-024-02038-0 (PMC11141006; doi:10.1186/s12912-024-02038-0)
Supplement: Supplementary file 1 — Supplementary Material 1. [file 12912_2024_2038_MOESM1_ESM.docx]

| **TS 1:** Extracted data | | | | | | | |
| --- | --- | --- | --- | --- | --- | --- | --- |
| **Authors/**  **Years/**  **Country** | **Purpose of study** | **Study design** | **Population** | **Sample size** | **Barriers** | **Positive Outcomes** | **Negative outcomes** |
| Younis et al., 2015  Egypt | To assess the effect of a planned TC  program on TC skills of paediatric nurses. | Quasi-experimental design | Paediatric nurses | 132 | - *Nurse-related barrier*   Younger age  Fewer years of practice experience  Lack of access to TC programme | Improvement in TC with patients, foster trust and improve recovery. | - |
| Amoah et al  2018  Ghana | To examine the perceived barriers to effective TC | Cross sectional | Nurses and patients | 112 (72 registered nurses and 40 patients) | - *Sociodemographic*   Language barrier,   - *Nurse-related barriers*   High workload, lack of knowledge on TC, use of jargons/technical terms, resistance and reluctance of the nurse to communicate, few years of experience   - *Patient-related barriers:*   Anxiety, pain, physical discomfort of the patient, lack of assurance of confidentiality, negative attitude of the patient towards the nurse, limited literacy   - *Environment-related:*   Unfamiliar environment, unsuitable environmental conditions (improper ventilation, heating, cooling, and lighting), busy environment of the ward,   - *Health system-related barriers:*   working multiple jobs, lack of enough time, being overworked during the shift | Adherence to treatment, patient involvement in their own care. | Dissatisfaction with care, misdiagnosis, misunderstanding, uncertainty, and frustration for both parties. |
| Cubaka et al. (2018)  Rwanda | To patients’ communication preferences and perceptions on what factors influence the patient–provider communication in primary health care setting | Qualitative | Patients | 16 OPD patients | - *Patient related factors:*   Limited literacy  Bad nursing care experience  Power imbalance weighing on the provider's side   - *Nurse-related factors:*   Providers' personality or mood,  inadequate knowledge  Oppressive or judgmental language   - *Health system-related barriers:*   Work overload,  Lack of communication training |  | - |
| Amoah et al.  (2019)  Ghana | To investigate the barriers to effective TC among patients and nurses | Qualitative | patients (admitted for 3-4days)  and nurses | 13 made up of:  6 nurses 7 patients. | - *Sociodemographic*   A*ge*: old age affects cognitive impairment that affects memory and comprehension  younger age comes with assumptions  Male Muslims do not prefer female nurses and vice-versa  cultural differences  patient status (socioeconomic status)  Language barrier   - *Patient-related characteristics*   idleness  patients’ misconception about nurses  pain   - *Nurse-related barriers*   High workload  lack of knowledge on TC  All knowing patients  families’ interference  Patient’s dissatisfaction   - *Environmental barriers*   anxiety related to new hospital environment  noisy environment  mosquito infestation in the health facility  extreme weather events  destructive environment (light, fan |  |  |
| Kambonde  2019  Namibia | To determine the factors that influence registered nurse-patient communication | Cross-sectional  survey | Registered nurses and patients | 159 (92 patients and 67 registered nurses | - *Health service factors:*   lack of orientation to the ward’s policies and procedures  Staff shortage,  Nurses’ workload   - *Nurse related factors:*   Stress (nurses),  Lack of self-introduction to patient (nurses),  Lack of skills | Patients’ satisfaction, patient compliance to treatment, | Non supportive management |
| Fite et al 2019  Ethiopia. | To identify factors that influence TC between nurses and patients | Cross-sectional study. | Patients (at least years and admitted for at least 3 days) | 200 | - *Sociodemographic*   Formal education, language difference, old age, sex difference   - *Patient related factors:*   presence of visitors, pain,   - *Nurses related factors:*   inadequate communication skills training, unfamiliar medical terms,   - *Health service-factors*:   nurses’ workload, lack of time, |  |  |
| Mohamed & Ahmed, 2019  Egypt | To determine the effect of nurse's TC and protecting patient's rights on patient's satisfaction | Cross-sectional survey | Nurses and Patients | 172 nurses and 200 patients | - *Health facility related factors:*   Patients in public facilities   - *Nurse-related factors*   Working in busy facility departments, and  longer years of working experience (> 10 years) | Patients’ satisfaction with nurses’ communication and protection of patients’ rights. |  |
| Wubneh et al., 2020  Ethiopia | To assess the level of the nurse-to-patient communication and perceived barriers. | Mixed-methods | Nurses | 387 (380 for the quantitative and 7 for the qualitative) | - *Patient– related barriers*   Presence of pain   - *Nurse-related:*   Communication barriers,   - *Environment-related barriers*   poor hospital environment   - *Health facility related*   high workload, |  |  |
| Arkorful et al  2021  Ghana | To explore barriers to effective TC between patients and nurses. | Qualitative | Nurses and patients (admitted for 5 days or more) | 60  30 each | - *Sociodemographic*   age: old & middle, Young  Religious influence (gestures and tenets)  Cultural practices & preferences  Male patients do not prefer female nurses and vice -versa  language barrier   - *Patient perceived barriers*   sense of professional decency and urgency of nurses, stereotypes (prejudice and malice) about nurses,  lack of respect for nurses   - *Nurses perceived barriers*   few nurses on the ward/human resource challenges, All knowing patients, patient lack of confidence in nurses’ competence, uncompromising/recalcitrant relatives, lack of knowledge on TC, patient satisfaction or vice versa with service, inability of nurses to explain technical issues, lack of empathy from nurses, pain, patients with special needs (eye, ear and speech impaired and the mentally challenged)   - *Health facility*   Prolong admission  Pressure on few staff   - *Environmental factors*   noise on the ward  mosquito infestation in the health facility  cold, hot and warm temperature.  destructive environment (light, fun and smell)  mood changes |  |  |
| Alqersh, 2021  Egypt | To evaluate the effect of infertility clinic nurse communication skills training on women satisfaction | Quasi-experimental design | Nurses and women seeking children. | 20 nurses and 200 women | Lack of access to communication skills education | Satisfaction with communication and treatment |  |
| Emishaw et al (2021)  Ethiopia | To explore the experience of nurses on perceived communication barriers. | Qualitative | nurses | 15 | - *Socio-demographic*   Older age of nurses & patients, younger nurses not interested in patients   - *Nurse related*   Physical fatigue of nurses  Lost energy  Lack of training   - *Patient-related*   Presence of pain  Family interference   - *Environment-related barriers*   Busy & noisy environment   - *Health-facility related*   Lack of continuous training opportunities  Lack of medical facilities and drugs  Ineffective health insurance practices |  |  |
| Appiah et al, 2022  Ghana | to assess effective nurse caregivers’ communication practices among pediatric nurses | Qualitative | paediatric nurses with at more than 2 years working experience | 43 | - *Nurse-related factors*   Nurses' attitude and behaviour   - *Patient-associated*   Negative patient attitude, deafness and anxiety  Negative attitude of patients' relatives. |  |  |
| Keutchafo et al., 2022  Cameroon | to explore factors that guide effective communication between nurses and older patients | Qualitative | Nurses and older patients | 34 older patients and 17 nurses | - *Sociodemographic*   Language barrier   - *Nurse-related*   negative stereotypes of elderly patients, compassionate care,   - *Health facility-related*   lack of long-term care facilities for the aged |  |  |
| Keutchafo et al., 2022  Cameroon | To understand the factors that solve communication problems between nurses and older patients. | Qualitative | Nurses | 17 nurses | - *Sociocultural:*   Culture believes that dying older patients are witches and wizards,   - *Nurse-related*   few years of working experience, nurses are unaware of non-verbal communication, lack of love for job,   - *Patient related factors*   when patients do not have money, and patients’ mood swings.   - *Health facility related*   high workload, |  |  |
| Othman et al., 2023  Egypt | To assess the effect of TC educational program for nurses on their nursing care quality | Quasi-experimental | Nurses | 50 | - *Nurse-related*   Lack of access to therapeutic educational programmes. |  |  |
| Appiah et al, 2023  Ghana | To explore the barriers to nurses' TC practice | Qualitative | professional nurses with at least 2 working experience | 30 | - *Patient-related factors*   Patient condition  Negative attitude patient  Family interference   - *Nurse-related factors*   Discriminatory attitude of nurses,  and stress.   - *Health facility factors*   High workload | Medical errors | Poor treatment compliance, safety concerns, patient dissatisfaction, and inefficient use of resources. |
